# Successful de-escalation antibiotic therapy using cephamycins for sepsis caused by extended-spectrum beta-lactamase-producing Enterobacteriaceae bacteremia: A sequential 25-case series

**DOI:** 10.1515/med-2020-0103

**Published:** 2020-08-07

**Authors:** Tsukasa Kuwana, Junko Yamaguchi, Kosaku Kinoshita, Satoshi Hori, Shingo Ihara, Tetsuya Taniguchi

**Affiliations:** Division of Emergency and Critical Care Medicine, Department of Acute Medicine, Nihon University School of Medicine, Tokyo, Japan; Division of Mathematics, Department of Liberal Arts, Nihon University School of Medicine, 30-1, Oyaguchi Kami-cho, Itabashi-ku, Tokyo 173-8610, Japan

**Keywords:** carbapenem, cefmetazole, cephamycin, ESBL-E bacteremia, de-escalation

## Abstract

Carbapenems are frequently used to treat infections caused by extended-spectrum beta-lactamase-producing Enterobacteriaceae (ESBL-E), but carbapenem-resistant Enterobacteriaceae bacteria are a clinical concern. Although cephamycins (cefmetazole; CMZ) have been shown to be effective against mild cases of ESBL-E infection, data on their use for severe ESBL-E infections with sepsis or septic shock remain scarce. Herein, we discuss a de-escalation therapy to CMZ that could be used after empiric antibiotic therapy in ICU patients with sepsis or septic shock caused by ESBL-E bacteremia. A sequence of 25 cases diagnosed with sepsis or septic shock caused by ESBL-E bacteria was evaluated. The attending infectious disease specialist physicians selected the antibiotics and decided the de-escalation timing. The median SOFA (Sequential Organ Failure Assessment) and APACHE II (Acute Physiology and Chronic Health Evaluation II) severity scores were 8 and 30; the rate of septic shock was 60%. Infections originated most frequently with urinary tract infection (UTI) (56%) and *Escherichia coli* (85%). Eleven patients were de-escalated to CMZ after vital signs were stable, and all survived. No patients died of UTI regardless of with or without de-escalation. The median timing of de-escalation antibiotic therapy after admission was 4 days (range, 3–6 days). At the time of de-escalation, the median SOFA score fell from 8 to 5, the median APACHE II score from 28 to 22, and the rate of septic shock from 55% to 0%. We conclude that for sepsis in UTI caused by ESBL-E bacteremia, de-escalation therapy from broad-spectrum antibiotics to CMZ is a potential treatment option when vital signs are stable.

## 1 Introduction

Infections caused by antimicrobial-resistant bacteria are a serious issue worldwide. Patients infected with extended-spectrum beta-lactamase-producing Enterobacteriaceae (ESBL-E) bacteria are often prescribed carbapenems [1]. As patients diagnosed with ESBL-E bacteremia have fewer options in terms of therapeutic antibiotics, carbapenems are frequently used throughout treatment [2]. The increased use of carbapenems increases the risk of resistant ESBL-E bacteria, and this might affect the incidence of carbapenem-resistant *Pseudomonas aeruginosa* or *Acinetobacter baumannii* [3].

Cephamycins (cefmetazole [CMZ], cefoxitin and cefotetan) structurally differ from other cephalosporins and have enhanced stability against ESBL. Although the administration of most cephalosporins for ESBL-E bacteremia often results in treatment failure or relapse, cephamycins such as cefotetan and cefoxitin are effective against more than 90% of ESBL-producing organisms [4]. CMZ is often used for community-acquired intra-abdominal infections of Enterobacteriaceae bacteria and anaerobes. While CMZ is stable against ESBL, it is generally not used as a first-line antibiotic therapy for sepsis caused by ESBL-E bacteremia in the world. Studies of alternatives to carbapenems [5,6] have shown no difference in the outcome between carbapenems and cephamycins as a definitive antibiotic therapy for ESBL-E bacteremia, but these studies did not evaluate the severe infection group. Moreover, their patients’ Sequential Organ Failure Assessment (SOFA) scores were low even if sepsis was present. Few studies have investigated the option of de-escalation to cephamycins after empiric antibiotic therapy in patients with sepsis caused by ESBL-E bacteremia and with higher SOFA scores. De-escalation therapy means switching from empirical anti-microbials to narrow spectrum anti-microbials against the identified causative bacteria.

In this case series, we report successful antibiotic de-escalation therapy and discuss whether de-escalation to cephamycins could be used after empiric antibiotic administration even in ICU patients with sepsis caused by ESBL-E bacteremia.

## 2 Case series report

We reviewed the cases of 28 patients who were sequentially admitted to the emergency critical care center between January 2008 and December 2017 and diagnosed with sepsis caused by ESBL-E bacteremia. Sepsis was defined according to the Third International Consensus definition, 2016 [7]. ESBL-E bacteria were isolated from initial blood cultures on admission in all cases. Microbiological identification was carried out using BACTEC™ FX (Becton, Dickinson and Company, Franklin Lakes, NJ, USA). From January 2008 to December 2013, ESBL production was evaluated by microdilution using the Dry Plate Eiken^®^ (Eiken, Tokyo, Japan) method, which was established by the Clinical & Laboratory Standards Institute [8]. From January 2014 to December 2017, ESBL production was evaluated using the Oxoid combination disk method with Mastdiscs AmpC & ESBL^®^ (Kanto Kagaku, Tokyo, Japan) [9].

Three end-stage chronic patients were excluded. The remaining 25 patients’ cases were reviewed retrospectively (Table 1). All patients were administered broad-spectrum antibiotics as an empiric antibiotic therapy after diagnosis of sepsis. Of these, 11 patients received de-escalation therapy using CMZ after the initial empiric antibiotic therapy, based on their blood culture report. For all 11 patients who received de-escalation therapy with CMZ, the CMZ susceptibility test for ESBL-E was susceptible. The 14 patients who received the same antibiotic throughout were classified as non-de-escalation patients. Nineteen (76%) patients survived, including all the de-escalation patients, and six (24%) died. No standard operating procedure was used regarding the timing and choice of antibiotics for de-escalation; antibiotic selection and de-escalation timing were decided by the attending infectious disease specialist physicians when vital signs were stable. The most common infection and causative organism were urinary tract infection (UTI) (56%) and *Escherichia coli* (85%), respectively. The median SOFA score was 8 (range, 7–11), median Acute Physiology and Chronic Health Evaluation II (APACHE II) score was 30 (range, 26–40), and rate of septic shock was 60% (15/25). The mortality rate was zero in UTI patients regardless of with or without de-escalation (Table 2).

**Table 1:** Treatments, characteristics, and outcomes for 25 patients with sepsis due to ESBL-E bacteremia

Case	Initial antibiotic (dose/day)	Susceptibility	SOFA	AKI	Source	Outcome
<De-escalation cases>						
1	Meropenem (3 g)	Susceptible	7	N	STI	Survived
2	Ceftriaxone (2 g)	Resistant	7	N	UTI	Survived
3	Tazobactam/piperacillin (9 g)	Susceptible	4	Y	UTI	Survived
4	Meropenem (3 g)	Susceptible	5	N	Pneumonia	Survived
5	Tazobactam/piperacillin (13.5 g)	Susceptible	8	N	Unknown	Survived
6	Tazobactam/piperacillin (18 g)	Susceptible	8	N	UTI	Survived
7	Tazobactam/piperacillin (18 g)	Susceptible	8	N	UTI	Survived
8	Tazobactam/piperacillin (13.5 g)	Susceptible	3	N	UTI	Survived
9	Doripenem (3 g)	Susceptible	10	N	Pneumonia	Survived
10	Doripenem (3 g)	Susceptible	14	N	UTI	Survived
11	Tazobactam/piperacillin (18 g)	Susceptible	11	N	UTI	Survived
<Non-de-escalation cases>						
1	Tazobactam/piperacillin (9 g)	Not tested^a^	15	Y	Pneumonia	Died
2	Ceftriaxone (4 g)	Resistant	4	N	UTI	Survived
3	Tazobactam/piperacillin (9 g)	Not tested^b^	12	Y	Unknown	Survived
4	Tazobactam/piperacillin (9 g)	Not tested^b^	10	Y	Pneumonia	Died
5	Doripenem (3 g)	Susceptible	8	N	UTI	Survived
6	Tazobactam/piperacillin (9 g)	Susceptible	9	Y	UTI	Survived
7	Tazobactam/piperacillin (6.75 g)	Susceptible	8	Y	UTI	Survived
8	Meropenem (2 g)	Susceptible	8	Y	Biliary tract	Died
9	Meropenem (3 g)	Susceptible	11	N	UTI	Survived
10	Tazobactam/piperacillin (18 g)	Susceptible	5	N	UTI	Survived
11	Tazobactam/piperacillin (18 g)	Not tested^b^	13	N	Pneumonia	Died
12	Cefepime (4 g) and gentamicin (300 mg)	Susceptible	23	Y	Unknown	Died
13	Doripenem (1.5 g)	Susceptible	4	N	UTI	Survived
14	Cefepime (2 g)	Susceptible	11	Y	Intra-abdominal	Died

Initial antibiotic: initial antibiotic use; Susceptibility: susceptibility to initial antibiotics; Y: yes; N: no; De-escalation: de-escalation to cephamycin (cefmetazole; CMZ); AKI: acute kidney injury; SOFA: Sequential Organ Failure Assessment score on admission; STI: soft tissue infection; UTI: urinary tract infection.

^a^No test was performed due to early death.

^b^Results are not available for three patients because there were no commercial kits until 2010.

**Table 2:** Baseline characteristics of patients in the de-escalation and non-de-escalation groups and patient mortality

	**All**	**De-escalation**		**Non-de-escalation**	
Number	25	11		14	
Age*	81 (69–85)	74 (64–85)		70 (61–80)	
Male	14 (56%)	6 (55%)		8 (57%)	
**Source**			**Mortality**		**Mortality**
UTI	14 (56%)	7 (64%)	0 (0％)	7 (50%)	0 (0%)
Pneumonia	5 (20%)	2 (18%)	0 (0%)	3 (21%)	3 (100%)
STI	1 (4%)	1 (9%)	0 (0%)		
Biliary tract	1 (4%)			1 (7%)	1 (100%)
Intra-abdominal	1 (4%)			1 (7%)	1 (100%)
Unknown	3 (12%)	1 (9%)	0 (0%)	2 (14%)	1 (50%)
**Pathogen (** ***n*** **= 26)**			**Mortality**		**Mortality**
*Escherichia coli*	22 (85%)	10 (1 case mixed)	0 (0%)	12	5 (42%)
*Klebsiella pneumoniae*	2 (8%)	2 (1 case mixed)	0 (0%)		
*Klebsiella oxytoca*	1 (4%)			1	1 (100%)
*Proteus mirabilis*	1 (4%)			1	0 (0%)
**Outcome**					
Survival in the ICU	19 (76%)	11 (100%)		8 (57%)	
**Patient profile**					
SOFA score*	8 (7–11)	8 (6–9)		10 (8–12)	
Respiration*	2 (2–3)	2 (2–2)		2 (2–4)	
Coagulation*	0 (0–1)	0 (0–1)		0 (0–3)	
Liver*	0 (0–0)	0 (0–0)		0 (0–1)	
Cardiovascular system*	1 (0–4)	3 (0–4)		1 (0–3)	
Central nervous system*	2 (1–3)	2 (1–3)		2 (1–3)	
Renal*	1 (0–4)	0 (0–1)		3 (1–4)	
APACHE II*	30 (26–40)	28 (24–33)		31 (28–44)	
Sepsis with shock	15 (60%)	6 (55%)		9 (64%)	
WBC (10^4^/µL)*	10 (7–20)	10 (7–18)		13 (7–28)	
CRP (mg/dl)*	9 (3–20)	9 (4–18)		11 (3–21)	
Initial antimicrobial susceptibility^a^	19/21	10/11		9/10	
CMZ resistance^b^	0	0		0	

Unmarked values: number of patients; *: median (interquartile range).

APACHE II: Acute Physiology and Chronic Health Evaluation II; CRP: C-reactive protein; ICU: intensive care unit; SOFA: Sequential Organ Failure Assessment; STI: soft tissue infection; UTI: urinary tract infection; WBC: white blood cell.

^a^Initial antimicrobial susceptibility test was performed for 11 patients in the CMZ group and 10 patients in the non-CMZ group.

^b^CMZ susceptibility test was performed in 11 patients in the CMZ group and 12 patients in the non-CMZ group.

The median timing of de-escalation antibiotic therapy after admission was 4 days (range, 3–6 days). At the time of de-escalation, the median SOFA score fell from 8 to 5 (cardiovascular element of the SOFA score was zero) and the median APACHE II score from 28 to 22 (Table 3). In conclusion, there was no case of persistent septic shock on day 4 after admission in the de-escalation patients, and no patient with septic shock who received treatment de-escalation to CMZ demonstrated recurrent shock thereafter.

**Table 3:** Comparison of characteristics at baseline vs time of de-escalation (de-escalation patients)

De-escalation patients* (*n* = 11)	On admission	Time of de-escalation
SOFA score	8 (6–9)	5 (4–7)
SOFA cardiovascular element	3 (0–4)	0 (0–1)
APACHE II	28 (24–33)	22 (19–25)
Sepsis with shock	6 (55%)	0 (0%)
WBC (10^4^/µL)	10 (7–18)	14 (9–17)
CRP (mg/dL)	9 (4–18)	9 (5–18)

*Results are shown as the median (interquartile range).

APACHE II: Acute Physiology and Chronic Health Evaluation II; CRP: C-reactive protein; SOFA: Sequential Organ Failure Assessment; WBC: white blood cell.

This study was approved by the Clinical Research Review Committee of the Nihon University School of Medicine (RK-180508-08). All patients received the document “Explanation and Consent Form Requesting Medical Research Cooperation”, and consent was obtained from each patient or his/her family by our institute.

## 3 Discussion

This case series suggests that de-escalation to cephamycins is a potential treatment option for patients with sepsis caused by ESBL-E bacteremia when vital signs are stable. In a previous study, CMZ was successfully used to treat ESBL-E bacteremia in patients with a low SOFA score (mean score, 2.8) [5]. In our study, the median SOFA score of the group of de-escalation patients was 8, including 60% patients with shock diagnosed according to the new sepsis definition [7]. In cases of severe infection suspected to be caused by ESBL-E bacteremia, the use of broad-spectrum antibiotics such as carbapenem from the beginning is recommended [10]. Even if ESBL-E bacteremia is confirmed, carbapenems are frequently continued, without de-escalation therapy [2]. There is no clear criterion for de-escalation therapy in such cases. While CMZ, which is classified as a cephamycin, does not affect ESBL-producers, it is theoretically useful against ESBL-E-associated infections. In this case series, all patients, excluding two patients who did not receive a CMZ susceptibility test due to early death, were susceptible to ESBL-E according to the CMZ susceptibility test. However, no clinical data have been reported on the effectiveness of cephamycins for ESBL-E-associated infections, excluding less severe bacteremia [5]. No studies have evaluated the outcome in ESBL-E bacteremia patients with septic shock on admission treated with CMZ after de-escalation. UTI caused by *Escherichia coli* is the most common infection, and it is the causative organism in ESBL-E bacteremia patients [2,5]. No patient in our cases had CMZ-resistant infection, and survival was 100% for patients who received CMZ as de-escalation therapy after initial empiric antibiotic therapy. Even if the sepsis severity score was high on admission, de-escalation therapy could potentially be considered a viable clinical option for patients who recovered from unstable circulation after the initial antibiotic therapy, especially in patients with UTI caused by *Escherichia coli*.

Although this case series demonstrates the importance of initial empiric antibiotic therapy for sepsis in UTI caused by ESBL-E bacteremia, no conclusion has been reached on whether de-escalation therapy is appropriate for sepsis. Because of the limited number of cases, we were not able to estimate statistically the difference between de-escalation and non-de-escalation patients. However, overuse of carbapenems for ESBL-E treatment is of critical clinical concern in relation to the increasing number of patients with carbapenem-resistant Enterobacteriaceae infection. According to the US Centers for Disease Control and Prevention, if the population of carbapenem-resistant bacteria among all Gram-negative bacilli increases in clinical settings, carbapenems might not be effective as an initial antibiotic therapy in the future [11]. Because novel antimicrobial drugs are rarely developed, it is important to reduce the use of carbapenems as much as possible to prevent tolerance [12]. The proportion of ESBL-E among the Enterobacteriaceae in intra-abdominal infections, not only in UTI, has been reported to have increased from 5.3% in 2010 to 13.5% in 2014 [13]. Against such a background, our case series potentially provides an optional de-escalation therapy for sepsis in severe ESBL-E infection.

There are some limitations in the results of this case series. For sepsis caused by ESBL-E bacteremia, de-escalation from broad-spectrum antibiotics to CMZ therapy exhibits potential as a useful treatment option. However, whether or not de-escalation antibiotic therapy is superior in terms of the final outcome for such patients remains unclear. There is a need for a controlled trial to address the issue of selection bias in the selection of initial antibiotics and timing of de-escalation, which is possible when patient numbers are limited.

## 4 Conclusions

For sepsis, especially in UTI cases caused by ESBL-E bacteremia, de-escalation from broad-spectrum antibiotics to cephamycin (specifically CMZ) therapy exhibits potential as a viable treatment option when vital signs are stable.

## References

[1] Paterson DL, Ko WC, Von Gottberg A, Mohapatra S, Casellas JM, Goossens H, et al. Antibiotic therapy for Klebsiella pneumoniae bacteremia: implications of production of extended-spectrum β-lactamases. Clin Infect Dis. 2004;39:31–7. 10.1086/420816. 15206050

[2] Harris PNA, Tambyah PA, Lye DC, Mo Y, Lee TH, Yilmaz M, et al. Effect of piperacillin-tazobactam vs meropenem on 30-day mortality for patients with E. coli or Klebsiella pneumonia bloodstream infection and ceftriaxone resistance: a randomized clinical trial. JAMA. 2018;320:984–94. 10.1001/jama.2018.12163. PMC6143100 30208454

[3] Falagas ME, Kopterides P. Risk factors for the isolation of multi-drug-resistant Acinetobacter baumannii and Pseudomonas aeruginosa: a systematic review of the literature. J Hosp Infect. 2006;64:7–15. 10.1016/j.jhin.2006.04.015. 16822583

[4] Paterson DL, Ko WC, Von Gottberg A, Casellas JM, Mulazimoglu L, Klugman KP, et al. Outcome of cephalosporin treatment for serious infections due to apparently susceptible organisms producing extended-spectrum b-lactamases: implications for the clinical microbiology laboratory. J Clin Microbiol. 2001;39:2206–12. 10.1128/JCM.39.6.2206-2212.2001. PMC88112 11376058

[5] Fukuchi T, Iwata K, Kobayashi S, Nakamura T, Ohji G. Cefmetazole for bacteremia caused by ESBL-producing enterobacteriaceae comparing with carbapenems. BMC Infect Dis. 2016;16:427. 10.1186/s12879-016-1770-1. PMC4991070 27538488

[6] Pilmis B, Parize P, Zahar JR, Lortholary O. Alternatives to carbapenems for infections caused by ESBL-producing Enterobacteriaceae. Eur J Clin Microbiol Infect Dis. 2014;33:1263–5. 10.1007/s10096-014-2094-y. 24691683

[7] Singer M, Deutschman CS, Seymour CW, Shankar-Hari M, Annane D, Bauer M, et al. The third international consensus definitions for sepsis and septic shock (Sepsis-3). JAMA. 2016;315:801–10. 10.1001/jama.2016.0287. PMC4968574 26903338

[8] Clinical and Laboratory Standards Institute. Performance standards for antimicrobial susceptibility testing; 19th informational supplement., M100-S19. Wayne, PA: Clinical and Laboratory Standards Institute; 2009.

[9] Carter MW, Oakton KJ, Warner M, Livermore DM. Detection of extended-spectrum β-lactamases in Klebsiellae with the Oxoid combination disk method. J Clin Microbiol. 2000;38:4228–32. 10.1128/jcm.38.11.4228-4232.2000 PMC87569 11060096

[10] Peleg AY, Hooper DC. Hospital-acquired infections due to gram-negative bacteria. N Engl J Med. 2010;362:1804–13. 10.1056/NEJMra0904124. PMC3107499 20463340

[11] Woodworth KR, Walters MS, Weiner LM, Edwards J, Brown AC, Huang JY, et al. Vital signs: containment of novel multidrug-resistant organisms and resistance mechanisms – United States, 2006–2017. MMWR Morb Mortal Wkly Rep. 2018;67:396–401. 10.15585/mmwr.mm6713e1. PMC5889247 29621209

[12] Ventola CL. The antibiotic resistance crisis: part 1: causes and threats. Pharm Ther. 2015;40:277–83. PMC4378521 25859123

[13] Takesue Y, Kusachi S, Mikamo H, Satoa J, Watanabea A, Kiyotaa H, et al. Antimicrobial susceptibility of common pathogens isolated from postoperative intra-abdominal infections in Japan. J Infect Chemother. 2018;24:330–40. 10.1016/j.jiac.2018.02.011. 29555391

